# Dissociation of liner from cup in THA: does liner damage affect the risk of dissociation?

**DOI:** 10.1007/s00402-022-04529-8

**Published:** 2022-07-05

**Authors:** Nicholas Andreas Beckmann, Mareike Schonhoff, Johannes Dominik Bastian, Tobias Renkawitz, Sebastian Jaeger

**Affiliations:** 1 Center for Orthopedics, Trauma Surgery and Spinal Cord Injury, Heidelberg University Hospital, Schlierbacher Landstrasse 200a, 69118 Heidelberg, Germany; 2 Laboratory of Biomechanics and Implant Research, Center for Orthopedics, Trauma Surgery and Spinal Cord Injury, Heidelberg University Hospital, Heidelberg, Germany; 3 Department of Orthopaedic Surgery and Traumatology, Inselspital, Bern University Hospital, University of Bern, Bern, Switzerland

**Keywords:** Dissociation, Liner, Hip, Complication, THA

## Abstract

**Introduction:**

A rare catastrophic failure of modular component Total Hip Arthroplasty is dissociation between liner and cup, which has been associated with component malposition and/or impingement and seems to be more frequently associated with the Pinnacle system. The goal of this study was to evaluate the resistance of a polyethylene liner to lever-out-forces of the Pinnacle locking mechanism and the locking mechanisms of two other current cup/liner systems using a standardized testing method (ASTM).

**Materials and methods:**

Five of each of the following cups were evaluated with their corresponding polyethylene liners: Pinnacle Multihole cup with and without intact anti-rotation tabs (ART’s); Allofit-S-Alloclassic and Plasmafit Plus7 cups. The ASTM test set-up was used to evaluate the lever-out force resulting in liner dissociation for each construct.

**Results:**

The Pinnacle construct with intact ARTs required the greatest force (*F*) to achieve dissociation (263.2 ± 79.2 N) followed by the Plasmafit Plus7 (185.8 ± 36.9 N) and the Allofit-S (101.4 ± 35.3 N) constructs, respectively. However, after removal of the ARTs, the Pinnacle system required the least force to achieve dissociation (75.1 ± 22.2 N) (*p* < 0.001).

**Conclusions:**

The intact Pinnacle system appeared the most stable in lever-out tests when compared to the other systems. However, after removal of the ARTs, the Pinnacle system required the least force for dissociation, consistent with locking mechanism failure, and suggesting that the ARTs are a critical component of the locking mechanism. Our findings are consistent with the clinical experience of dissociated Pinnacle constructs displaying damaged or missing ARTs, and that damage to these may increase risk of liner dissociation.

## Introduction

**Fig. 1:**
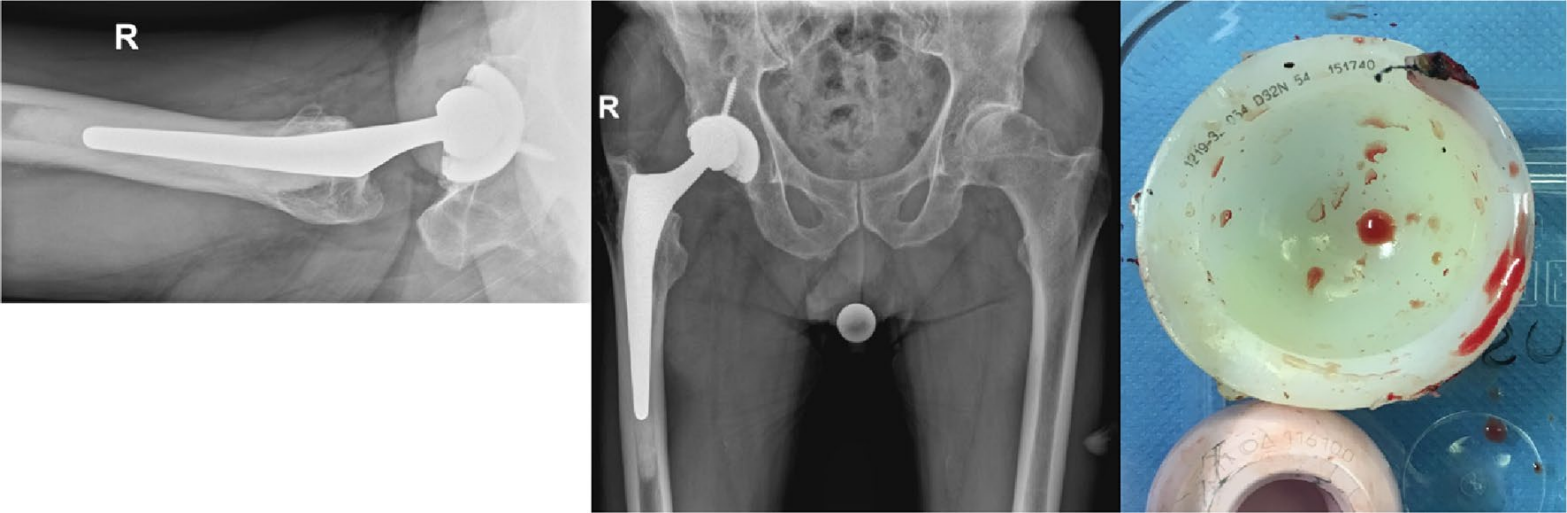
Example of dissociated Pinnacle liner from a patient we had treated (left axial hip X-ray; center low ap pelvis X-ray; right photograph after removal of liner with four damaged or missing anti-rotation tabs at time of removal)

Component modularity in Total Hip Arthroplasty (THA) has facilitated increased surgical flexibility, allowing improved component positioning and conservative revisions [5]. However, a rare but catastrophic failure of modular component hip surgery is dissociation between liner and cup as previously described in the 1990s with the Polyethylene liner in Harris-Galante implants [8]. Since then, there have been infrequent reports of a number of different cup/liner combinations that have dissociated [15, 21], but most have involved the Pinnacle system (DePuy Synthes, Warsaw, IN, USA) (example Fig. 1), generally with a Polyethylene liner [21, 31, 36]. The incidence is low, with some authors reporting a frequency of 0, 8% [36], but it may be underreported [21]. Several predisposing factors, including component malposition and/or impingement, have been suggested for this complication as a result of a failure of the unique locking mechanism [21].

Standardized tests have been developed to evaluate cup/liner locking mechanisms (LM) including the axial (push out), lever out, and torque disassembly tests (Standard Test Method for Determining the Forces of Disassembly of Modular Acetabular Devices-F1820-13 (ASTM) [7]). The lever-out test was developed “to model the physiologic loading conditions present in the extremes of hip flexion and extension as well as situations of variable head coverage” [32]. This test mimics the forces that occur when the neck of a femoral prosthesis impinges on the edge of the liner [18] and could cause the edge of the acetabular liner opposite to the area of impingement to be pushed out of the shell (ASTM F1820-13) [7, 18].

The goal of this study was to evaluate the resistance to lever-out forces of several distinct acetabular cup and liner designs [13] (Pinnacle^®^ cup with Marathon^®^ liner as well as two other currently used systems) using a standardized test method (ASTM standard F1820-13) [7]. The Pinnacle^®^ system was evaluated intact and after removal of the six anti-rotation tabs (ARTs), since cases of Pinnacle^®^ liner dissociation have frequently been associated with the loss of three or four of these tabs [9, 11, 18–22, 31, 36]. Current results were compared to those of our prior push-out test that used the same constructs [13].

We hypothesized that the Pinnacle/Marathon construct with and without the ARTs would demonstrate a weaker LM than the other constructs.

## Materials and methods

We evaluated four groups (A, B, C, D) with three different acetabular cup/polyethylene (PE) liner systems and interface geometries. These are the most commonly used systems according to EPRD 2021 registry with distinctly different LM, and each with a sample size of five (see details below) [10]. All 20 cup/liner samples were subjected to lever out disassembly forces utilizing the standardized ASTM International method (F1820-13) [7] and tested once only [24, 32].

**Fig. 2:**
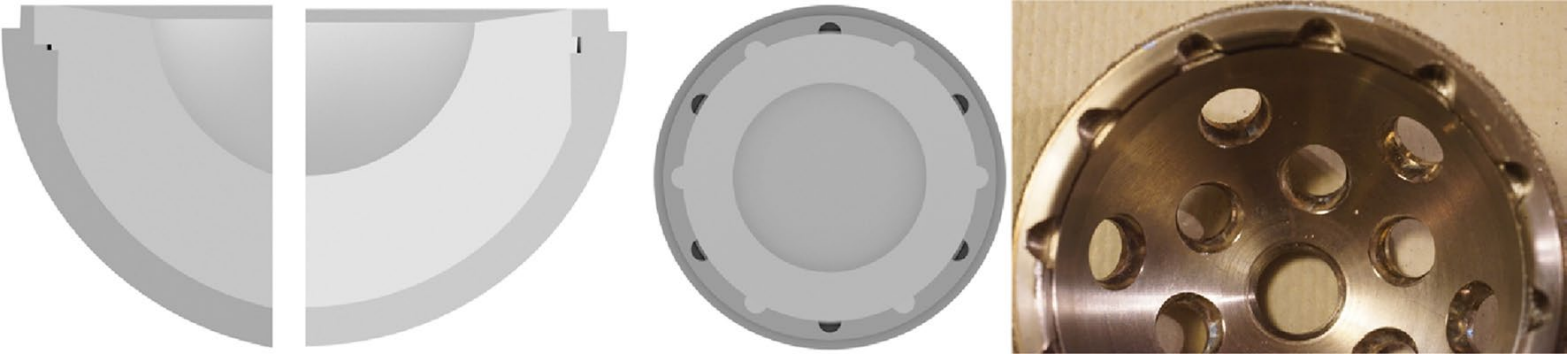
From left to right: Schematic Computer-Aided Drawing (CAD) showing a cross-section of the Pinnacle cup and liner with ART; CAD showing a cross-section of the Pinnacle cup and liner between ARTs; CAD axial view of the Pinnacle cup with liner; Photograph of Pinnacle cup

*Group A:* Pinnacle Multihole cup with moderately cross-linked Marathon liner (DePuy Synthes, Warsaw, IN, USA). The cup has a central dome region and LM consisting of a shallow circumferential groove that accommodates a small, raised ring on the liner and a short 10-degree Morse taper. The cup also has 12 scallops that extend to the rim, that accept six anti-rotation tabs on the liner. After seating, the liner sits approximately 1 mm proud of the metal cup rim [11, 15, 19]. The concave surface of the cup is smooth with a roughness of Ra = 0.2 μm and Rz = 1 μm (see Fig. 2).

**Fig. 3:**
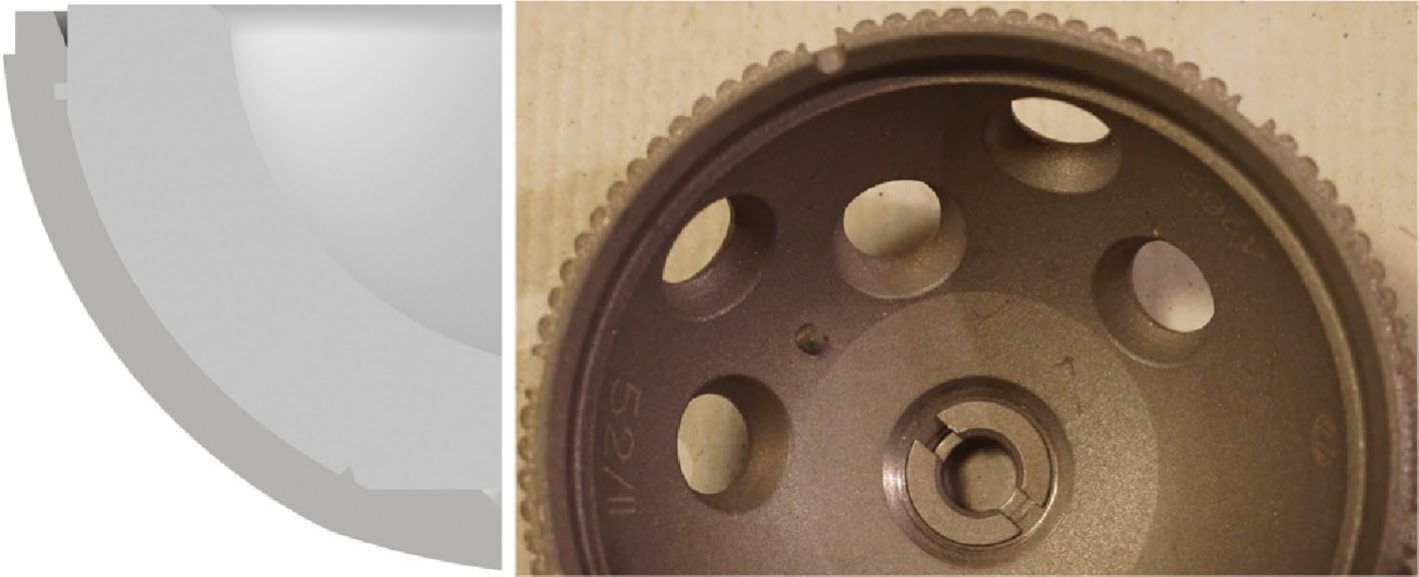
CAD image showing a cross-section half of the Multihole Allofit cup with liner (left); Photograph of Allofit cup (right)

*Group B:* Allofit-S Alloclassic with a highly cross-linked Durasul liner (Zimmer Biomet, Warsaw, IN, USA). The LM entails a circular press-fit with an additional two metal spikes at the inner polar region of the cup that insert into the liner for additional rotational stability. The concave cup surface is smooth with a roughness of Ra = 1 μm; Rz = 6–8 μm (see Fig. 3).

**Fig. 4:**
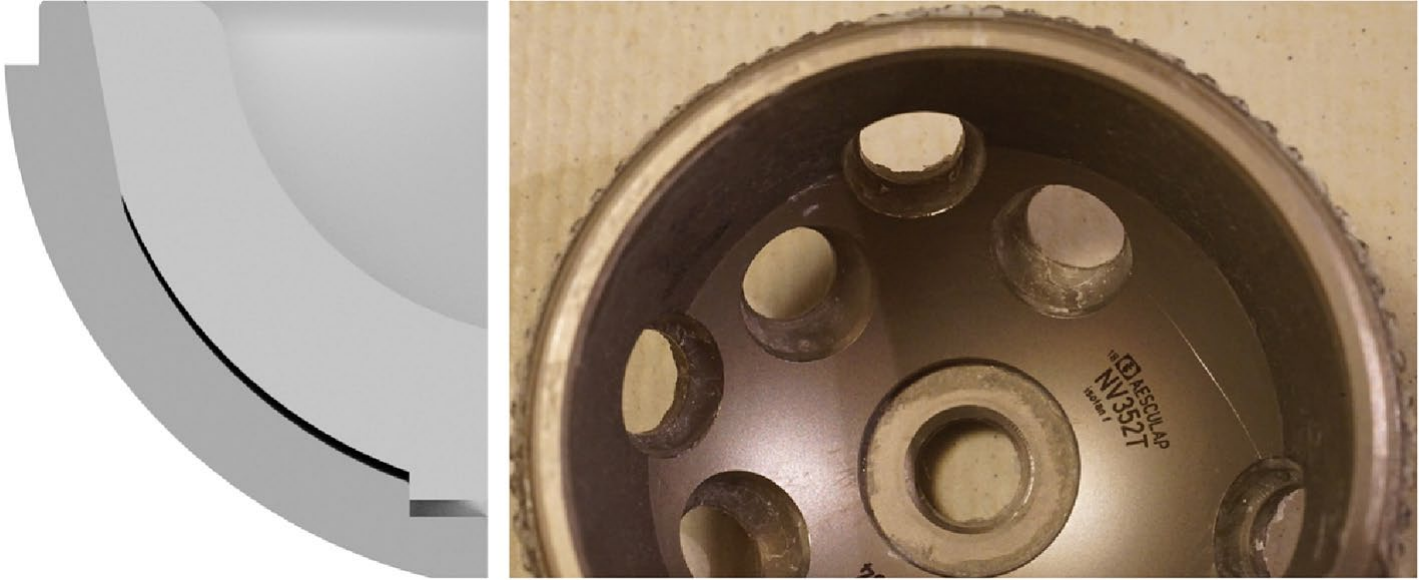
CAD image showing one cross-section of half of the Plasmafit cup with liner (left); axial Photograph of the Plasmafit cup (right)

*Group C:* Plasmafit^®^ Plus7 cup with a conventional UHMWPE liner (Aesculap, Tuttlingen, BW, Germany) is fixed by a conical LM. The concave surface of the cup is rough with a roughness of Ra = 4 μm, Rz = 25 μm. There is a cylindrical closing plug at the pole of the liner which extends into an indentation at the center of the dome of the cup (see Fig. 4).

**Fig. 5:**
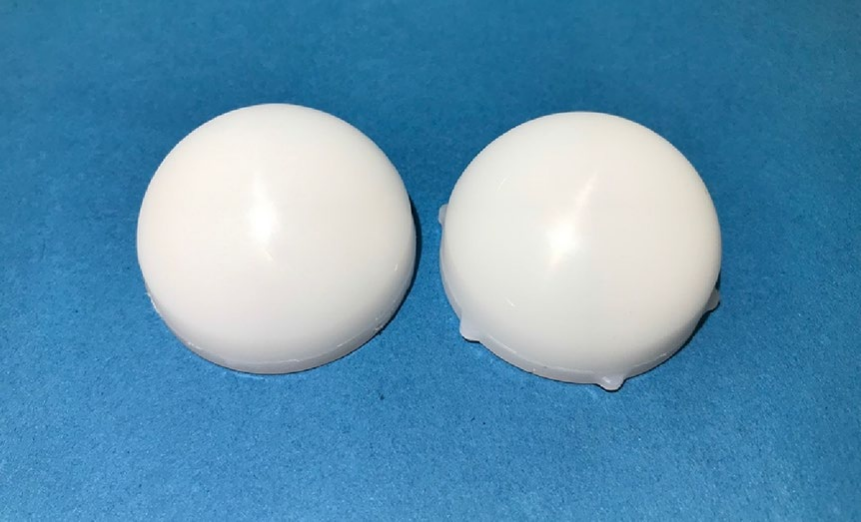
3D CAD image showing the Pinnacle liner [left with the anti-rotation tabs (ARTs) removed; on right ARTs intact]

*Group D:* Same as A, however, the anti-rotation tabs (ARTs) were removed by shearing them off with a scalpel to the level of the remaining liner; the circumferential lip was left intact (see Fig. 5). This was done to mimic the loss of ARTs noted during prior revisions for Pinnacle liner dissociation and to assess the ART contribution to the LM. Detailed illustrations and descriptions of these locking mechanisms and implants were published in Jaeger et al. [13].

All of the above were non-cemented cups with a 52 mm outer diameter and 32 mm neutral press-fit liner.

Prior to testing, each liner was press-fit into the respective cup by the application of a 2kN force applied coincident with the polar axis of the liner [7] and each liner had the creation of a rectangular slot on the inner surface at 80% of the height (*h*_1_) of the polar axis of the liner (*h*). The location of the slot was separated from the locking mechanism.

**Fig. 6:**
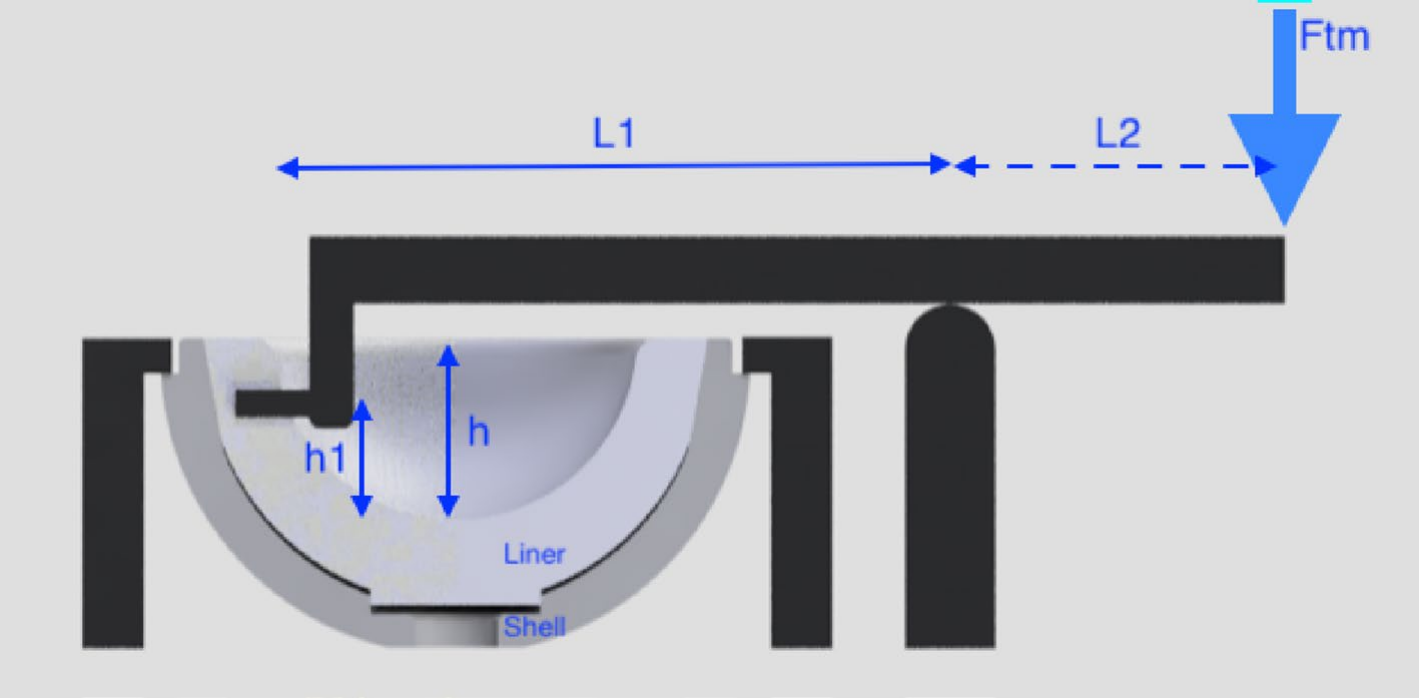
Schematic drawing of the test set-up testing a Plasmafit^®^ Cup and Liner (group C)

The cup/liner construct was firmly clamped in a secure fixture, facing upwards, and with the liner unrestricted by the fixation device (Fig. 6). The implantations and testing were carried out by an attending/consultant orthopedic surgeon and engineers (NAB, SJ, and MS).

A leverage device was set up, with the lever parallel to the surface of the cup/liner and with a lever arm offset that fitted into the liner slot, as shown in Fig. 6. The lever fulcrum point was placed adjacent to the cup/liner construct and at a distance of *L*_1_ (60 mm) from the lever offset into the liner slot. The force was applied using a material testing machine (Zwick Roell, Z005, Ulm, Germany) at a distance of *L*_2_ (70 mm) from the fulcrum point, and at a traverse speed of 5.1 cm/min. Disassembly of the system was defined by separation of the liner from the cup and/or loss of resistance to lever out, which was confirmed visually and through computer data. The required force for disassembly was recorded.

Distances *L*_1_ and *L*_2_ and the disassembly force were measured and the force, *F*, necessary for disassembly was calculated using: *F* = F_tm_ × (L_2_/L_1_), where *F*_tm_ = the force reading on the testing machine.

### Statistics

The sample size (*n* = 5/group) was determined by the ASTM norm, so that a pre-testing sample size estimation was not necessary [7].

A one-way ANOVA was performed to assess the effect of cup design on the maximum lever-out force. To identify the differences between pairs of means that were significant, a post hoc analysis was performed. We used a Bonferroni test as a post hoc analysis to explore differences between the four group means while controlling the experiment incurred error rate.

Normal distribution of the data was evaluated using a Shapiro–Wilk test and the homogeneity of variance was verified using the Levene test. The results allowed for the use of the ANOVA test.

Additionally, the data were evaluated descriptively using the arithmetic mean, standard deviation, minimum, and maximum. The data were analyzed using SPSS 25 (IBM, Armonk, NY, USA) with a significance level of *p* < 0.05.

## Results

**Fig. 7:**
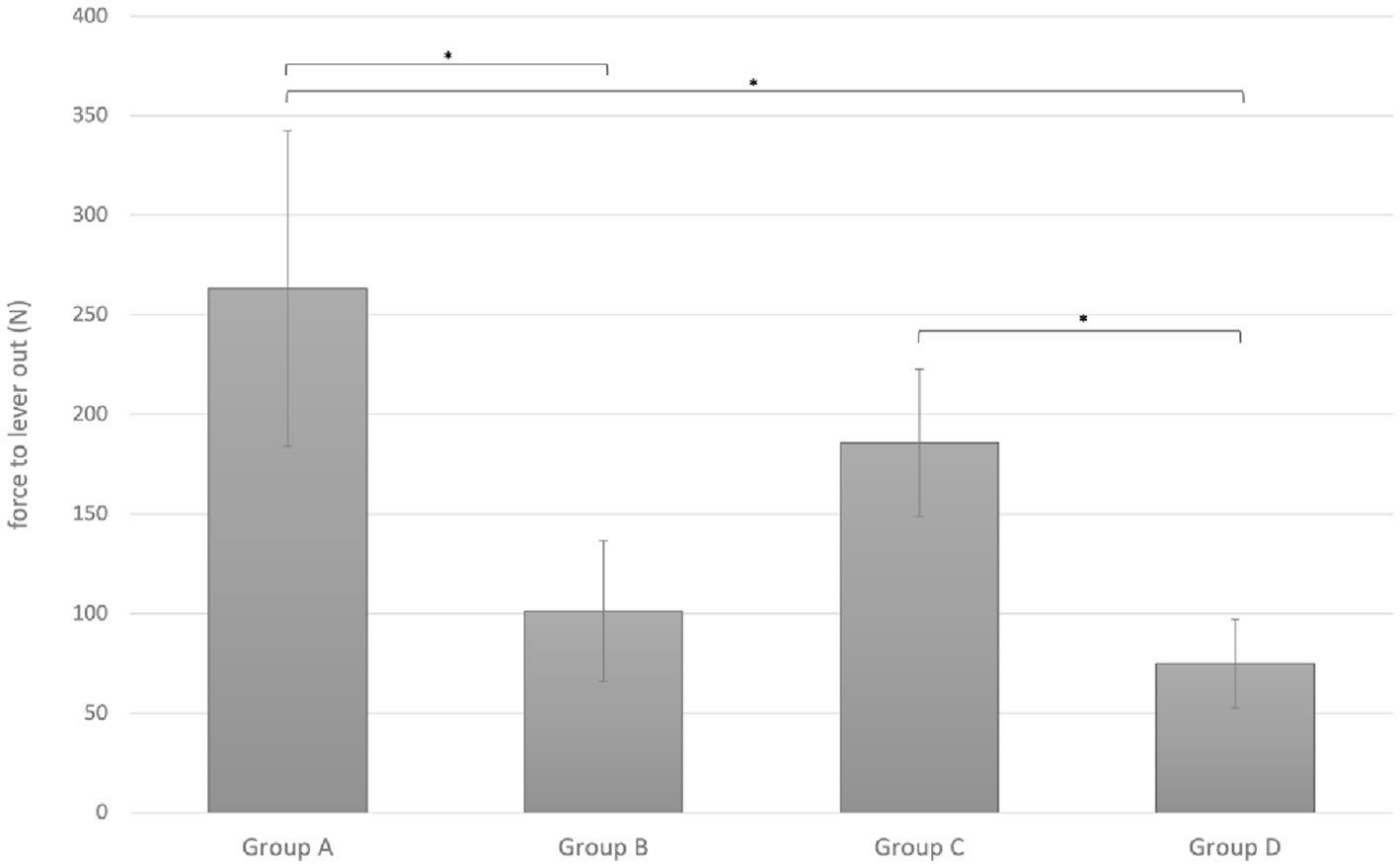
Mean (and SD) force to liner lever-out for respective groups (significant differences between groups denoted by*)

The lever-out forces that resulted in liner disassembly for each of the four groups are shown in Table 1. The greatest force had to be applied to the DePuy Synthes Pinnacle^®^ Multihole system followed by the Aesculap Plasmafit^®^ Plus7 implant. The least force was recorded for the Zimmer Biomet Allofit^®^-S Alloclassic^®^ implant. However, after removal of the anti-rotational tabs from the Pinnacle liner, the lever-out force to achieve separation of cup and liner was lower for the Pinnacle system than for all other constructs. These findings are demonstrated in Fig. 7.

**Table 1:** Lever-out Force, *F* (N), calculated according to ASTM using *F* = Ftm × (L2/L1)

	Group A	Group B	Group C	Group D
	Pinnacle^®^-Multihole	Allofit^®^-S Alloclassic^®^	Plasmafit^®^ Plus7	Pinnacle^®^-Multihole without anti-rot. tabs
Mean	263.2	101.4	185.8	75.1
SD	79.2	35.3	36.9	22.2
Min	169.2	67.2	116.7	59.4
Max	336.0	158.7	199.5	114.1

One-way ANOVA revealed a significant difference (*p* < 0.001) between the means of the four groups. The post hoc analysis revealed that the lever-out forces were statistically significantly different between the intact Pinnacle system (group A) and the Allofit system (group B), between the Pinnacle implants with anti-rotational tabs (group A) and those without the anti-rotational tabs (group D) and between the Plasmafit (group C) and the Pinnacle implants without tabs (group D). No significant differences were noted between the intact Pinnacle system and the Plasmafit system, between the Allofit and Plasmafit system and Allofit and Pinnacle system without Tabs (Table 2).

**Table 2:** Statistical significance, *p* value, of differences between the respective groups (*p* < 0.05 was considered significant (*))

Group comparison		*P* value	
Group A	Group B	0.001	*
Group A	Group C	0.129	
Group A	Group D	< 0.001	*
Group B	Group C	0.080	
Group B	Group D	1.000	
Group C	Group D	0.013	*

## Discussion

Component modularity in THA allows increased surgical flexibility, but with the risk of the rare occurrence of separation between liner and cup (liner dissociation). Historically liner dissociation was attributed to PE liner wear and LM failure of the first-generation Harris-Galante constructs [8, 21, 28, 35]. Other constructs that are less frequently used in Germany and have dissociated include Trident (Stryker), Furlong CSF (JRI Orthopaedics), and Trilogy (Zimmer) [15, 21]. The occurrence of dissociation led to the development of tests to assess LM integrity and resistance to disassembly [7, 32], and ultimately to the development of cross-linked PE and improved component design.

Liner disassembly has been reported rarely in the third-generation cups, but is most commonly associated with the Pinnacle cup system, with Enduron, Marathon [21] and AltrX liners [21] despite an excellent 10 year survival for any cause of up to 99.2% [2, 4]. The reported rates of Pinnacle liner dissociation vary from 0.07 [14, 21] to 0.82% [36]. Dissociation and dislocation have similar risk factors and may co-exist clinically [21], possibly resulting in under-reporting of dissociation in registry data [24].

Several tests to assess the LM have been used for the Pinnacle system. Push-out and lever-out forces have been used by Postak et al. who conducted a comparative evaluation of Pinnacle with Enduron and Marathon liners and two other comparable cup systems [25]. The Pinnacle system required the least force for dissociation; however, the standardized ASTM methodology was not used. Jaeger et al. utilized ASTM methodology and performed push-out tests on Pinnacle with Marathon liner and compared it to the Biomet and Aesculap constructs [13]. Pinnacle performed more poorly than the others. However, this test does not recruit ART function, since no torsion/rotation is incurred. Backside relative micromotion between cup and liner was also measured, and Pinnacle micromotion was in line with others. Backside micromotion was not eliminated in these third-generation cups, but it is unclear how it relates to liner dissociation. Push-out disassembly tests fail to simulate physiologic loading. Lever-out tests more closely simulate the torque force acting on the liner during the extremes of hip range of motion (ROM) [32]. The lever-out test of the ASTM method is designed to assess the integrity of the LM to withstand edge forces of impingement. Impingement can cause the edge of the liner opposite to the site of impingement to disengage from the shell and be pushed out [7] as a result of the femoral head moving up the rim of the liner. The same mechanism can lead to femoral head subluxation [23, 27, 33].

Our lever-out test used ASTM methodology and showed that the Pinnacle/Marathon construct with intact ARTs performed better than Biomet and Aesculap constructs. However, removal of the Pinnacle ARTs caused a significant deterioration in performance compared to the intact Pinnacle system although not significantly different from the Zimmer Biomet construct (Tables 1, 2, Fig. 7). Our results demonstrated the critical role of the ARTs in the integrity of the Pinnacle LM. Operative findings during revision surgery for dissociated Pinnacle^®^ liners showed multiple ART loss (at least three tabs) with liner rotation and displacement [3, 15, 18–22, 31]. The location of tab loss was usually not specified, although a superior location was occasionally reported, consistent with the area of maximal liner wear that occurs under circumstances of normal activity [13, 34].

Clinically, the time from surgery to liner dissociation has been considered early or late, before or after 2 years, respectively [3, 9, 18–21]. Early cases have been more frequently associated with cup malposition and liner mal-seating, later cases with extremes of ROM in addition to cup malposition [18]. Yun et al. had 21 cases of liner dissociation; 7 cases with offset face-changing liners had a mean time from surgery to dissociation of 12.7 months compared to 14 cases with neutral liners that dissociated 66 months post-surgery. Of the 7 cases, 5 had an increased abduction angle versus only 1 of the latter 14 cases. Cup malposition has frequently been cited as a risk factor for impingement and liner dissociation [9, 20, 21, 36], but liner dissociation has also been reported in cases with no apparent cup malposition [11, 15, 21, 22, 36].

Impingement and/or edge loading have been implicated as causative in almost all cases of liner dissociation [18, 20–22, 36] in addition to causing increased component wear, loosening, and decreased ROM [27]. Impingement has been shown to occur more frequently than clinically indicated [29, 33], more frequently in men in one study [27], and in patients with no history of cup malposition [29]. Shon conducted retrieval studies of acetabular components for evidence of impingement; 41% with no history of dislocation had liner evidence of impingement [23]. It has been suggested that some liner damage resulted from femoral head contact against the rim [29], consistent with secondary edge loading or contrecoup force opposite to the impingement site [18]. Usrey et al. suggested that during impingement, the liner rim at the impingement site acts as a fulcrum to lift and move the femoral head up the opposite side of the liner wall [33]. Impingement has also been associated with pelvic tilt variations [26, 30].

Extremes of ROM (squatting and kneeling) may cause impingement with a contrecoup leverage force of the femoral head on the liner [17, 18, 21, 29, 33], or edge loading in the absence of impingement [12].

Primary edge loading without impingement may also occur with cup malposition. It has been predicted to occur with cup inclination angles ≥ 55 degrees during cycles of normal daily activities (walking, ascending/descending stairs) in finite-element studies [12]. Edge loading studies have predicted a substantial increase in stress and plastic strain of the PE [12].

We hypothesize that the mechanism underlying dissociation differs with different clinical scenarios and risk factors. We propose that impingement is the predominant cause in cases of mal-seating and cup malposition, particularly in early dissociation, and can occur anywhere on the liner rim, but is most common posteriorly [29]. At the locus of impingement, the liner LM is subjected to increased sheer stress and torsion [1]. In addition, the site opposite to the impingement site is subject to a contrecoup leverage force as a result of the femoral head sliding along the rim, with possible subluxation [7, 18, 19, 24, 29]. Cases with severe cup abduction have additional stress on the liner [6]. This scenario results in two areas of LM and ART damage.

In late cases, extremes of ROM tend to be more frequent than in early cases and cause the femoral head/liner contact patch to move toward the rim [12] constituting edge loading. Associated age-related PE deterioration of the liner LM [24] can result in local LM failure and incongruity of liner/shell contact. This allows increased liner motion/rotation under loading force with damage and shearing off of the ART’s, with further liner rotation and dissociation [15, 16, 18, 31]. If impingement also occurs, there is coincident damage at the impingement site.

In clinical studies, only the liner component of the LM is damaged in most cases [11, 21, 36]. It is thought that the design of the LM (Morse taper, ridge, and groove) is a major contributor to failure. Age deterioration of the PE may also play a role [24]. We demonstrated that the LM performed poorly without ARTs, indicating that they have a critical role in the stability of the LM. We propose that the initiating event is damage to and failure of the LM that subjects the ARTs to increased torsional stress and causes them to be sheared off, allowing rotation and complete dissociation.

Preventive measures include the avoidance of large femoral head/thin PE rim combinations, careful liner seating with no interposing material, and optimal cup orientation with intra-operative confirmation of no impingement. Patients should minimize extremes of ROM (squatting, kneeling, and sitting cross-legged) and to seek help for hip pain, instability, or joint squeaking.

### Limitations

Only one type of Pinnacle liner was used. Results may differ with use of Enduron or AltrX liners.

Our study utilized only two types of cup/liner constructs other than Pinnacle/Marathon. Our study does not evaluate the effects of head size and/or liner thickness on liner dissociation.

The ASTM methodology was closely followed to simulate the effect of impingement on the liner and indicate the disassembly force resulting from it. However, since no femoral head was present, the in vivo effect of femoral tilt, combined anteversion of the femoral component and stem [23, 26], or the contrecoup effect of impingement, could not be simulated.

Because this was an in vitro set-up with all cups secured in a standardized manner, the properties of the fixation method did not correspond to the in vivo scenario of live bone and surrounding soft tissue.

All liners were press-fit into cups with 2kN impaction force, as specified in the ASTM standard, which may not reflect the clinical surgical situation.

## Conclusion

In our biomechanical study, we evaluated the liner/cup locking mechanism in three different implant systems. The intact Pinnacle system appeared to be the most stable in lever-out tests unless there was damage to or absence of the ARTs, which then resulted in the least stability to lever out force. The four groups had a large variation in standard deviation, indicating large variation in implant stability between samples. Our results showed that the ARTs of the Pinnacle^®^ system were critical to the integrity of the locking mechanism. This is consistent with the surgical findings of sheared off ARTs at revision surgery for Pinnacle^®^ liner dissociation.

Our biomechanical study indicates that liner dissociation results from failure of the locking mechanism secondary to impingement and/or edge-loading that is frequently associated with component malposition and/or extremes of range of motion. Therefore, component malposition should be carefully avoided during surgical intervention.

The Pinnacle system has been widely used with up to 99.2% 10 year survival from any cause [4]. Although more cases of dissociation have been reported with Pinnacle than with other systems, dissociation remains a rare event and the incidence can be reduced further with optimal surgical technique.
